# The effect of pelvic physiotherapy on reduction of functional constipation in children: design of a multicentre randomised controlled trial

**DOI:** 10.1186/1471-2431-13-112

**Published:** 2013-08-02

**Authors:** Marieke L van Engelenburg – van Lonkhuyzen, Esther MJ Bols, Marc A Benninga, Wim A Verwijs, Netty MWL Bluijssen, Rob A de Bie

**Affiliations:** 1 Department of Epidemiology, School for Public Health and Primary Care (CAPHRI), Maastricht University Medical Centre (MUMC+), PO Box 616, 6200, MD, Maastricht, the Netherlands; 2 Centre for Evidence Based Physiotherapy, MUMC+, PO Box 616, 6200, MD, Maastricht, the Netherlands; 3 Department of Paediatric Gastroenterology, Emma Children’s Hospital/AMC, Meibergdreef 9, 1105, AZ, Amsterdam, the Netherlands; 4 Zuwe Hofpoort Ziekenhuis, Polanerbaan 2, 3447, GN, Woerden, the Netherlands; 5 Fysiotherapie De Groote Wielen, Grootewielenlaan 95, 5247, JA, Rosmalen, the Netherlands

**Keywords:** Biofeedback, Children, Constipation, Incontinence, Manometry, Pelvic floor, Physiotherapy, Physical therapy, Randomised controlled trial, Study protocol

## Abstract

**Background:**

Functional constipation is a common disorder worldwide and is found in all paediatric age groups. Functional constipation can be caused by delayed colonic transit or dysfunction of the pelvic floor muscles. Standard medical care in paediatric practice is often based on clinical experience and mainly consists of a behavioural approach and toilet training, along with the prescription of laxatives. Evidence to evaluate the effectiveness of pelvic physiotherapy for this complaint is lacking.

**Methods/design:**

A two-armed multicentre randomised controlled trial has been designed. We hypothesise that the combination of pelvic physiotherapy and standard medical care will be more effective than standard medical care alone for constipated children, aged 5 to 17 years. Children with functional constipation according to the Rome III will be included. Web-based baseline and follow-up measurements, scheduled at 3 and 6 months after inclusion, consist of the numeric rating scale in relation to the perceived severity of the problem, the Strength and Difficulties Questionnaire and subjective improvement post-intervention (global perceived effect). Examination of the pelvic floor muscle functions, including digital testing and biofeedback, will take place during baseline and follow-up measurements at the physiotherapist. The control group will only receive standard medical care, involving at least three contacts during five months, whereas the experimental group will receive standard medical care plus pelvic physiotherapy, with a maximum of six contacts. The physiotherapy intervention will include standard medical care, pelvic floor muscle training, attention to breathing, relaxation and awareness of body and posture. The study duration will be six months from randomisation, with a three-year recruitment period. The primary outcome is the absence of functional constipation according to the Rome III criteria.

**Discussion:**

This section discusses the relevance of publishing the study design and the development of the presented physiotherapy protocol. It also addresses difficulties when interpreting the literature with regard to the effectiveness of biofeedback, potential confounding, and future research indications. To our knowledge, this article is the first to describe the design of a randomised controlled trial among children with constipation to assess the effect of pelvic physiotherapy as an add-on to standard medical care.

**Trial registration:**

Current Controlled Trials NL30551.068.09

## Background

Constipation is a common disorder worldwide, affecting 0.7% to 29.6% (median 12%) of children aged 0–18 years, and is found in all paediatric age groups with a severity that ranges from mild to severe and a duration that ranges from brief to chronic [1-4]. Symptoms of constipation are included in the Rome III criteria (Table 1), which were formulated by paediatric gastroenterologists in 2006 [3,5]. Constipation has a major impact on a child’s psychosocial functioning. About 40% of these children are burdened by emotional problems, such as eating disorders, truancy, family problems, social isolation and depression [6-10].

**Table 1:** Paediatric Rome III criteria* for childhood constipation

**Must include two or more of the following in a child with a**	
**developmental age of least four years**	
1	Two or fewer defaecations in the toilet per week
2	At least one episode of faecal incontinence per week
3	History of retentive posturing or excessive volitional stool retention
4	History of painful or hard bowel movement
5	Presence of a large faecal mass in the rectum
6	History of large diameter stools that may obstruct the toilet

*Criteria fulfilled at least once per week for at least two months prior to diagnosis, with insufficient criteria for diagnosis of irritable bowel syndrome.

The pathophysiology of childhood constipation is multifactorial and not yet fully understood. No clear organic cause is found in over 90% of the children who suffer from constipation, so-called functional constipation (FC). FC includes delayed colonic transit and dyssynergic defaecation. In the majority of children dyssynergic defaecation is the cause for their complaints, and refers to an incomplete evacuation of faeces due to paradoxical contraction of, or failure to relax the pelvic floor muscles when straining and/or a failure to increase intra rectal pressure [11,12]. In some patients, delayed colonic transit may be the result of dyssynergic defaecation. Approximately 50% of children show this abnormal defecation pattern [13]. Withdrawal of stool, hard painful defecation or fear of stool, resulting in a vicious circle, is the most commonly proposed explanation of the aetiology of dyssynergic constipation in children [4,7,12,14-16].

The standard medical care (SMC) for FC children in routine paediatric practice is often based on clinical experience and mainly consists of a behavioural approach and toilet training along with the prescription of laxatives, sometimes supported by disimpaction or a high fibre diet [7,10,14,17,18]. Despite intensive medical and behavioural treatment long-term follow-up studies have shown that 50% of the children still have complaints of constipation after five year follow up [19].

Since dyssynergic dysfunction of the pelvic floor is the major reason for FC in children, it is thought that pelvic physiotherapists, who have specific expertise in treating dyssynergic dysfunction, as musculoskeletal experts might play an important role to increase the success rate. All prerequisites for proper micturition and defecation, such as a relaxed toilet posture [18,20], adequate straining [18,21,22] and trunk stability [23-26], are trained extensively. Pelvic floor muscle training involves training the right use of the pelvic floor muscles (PFM) during contracting and straining, breathing and changes in abdominal pressure [21,22,27]. In case of (urine) incontinence, the treatment can be extended with additional PFM exercises.

Standard pelvic physiotherapy (PPT) care for the constipated child has not been described. In most cases, PPT consists of education, demystification, the use of micturition and defecation diaries, toilet training, breathing and relaxation exercises and pelvic floor muscle training (PFMT), with PFMT including exercises and biofeedback (BF) [28,29].

## Methods/design

### Trial design, hypothesis and research

To date, there has been little scientific evidence evaluating the effectiveness of pelvic physiotherapy (PPT) in childhood constipation [7,14,29,30]. Therefore we designed a two-armed multicentre randomised controlled clinical trial (RCT). We hypothesise that the combination of standard medical care (SMC) and PPT will prove more effective than SMC alone for the treatment of FC in children aged 5 to 17 years.

Secondary research questions are: (1) Does PPT add to the child’s quality of life, as defined by the strengths and difficulties questionnaire (SDQ), the numeric rating scale (NRS) in relation to the perceived severity of the problem and the global perceived effect (GPE), (2) what values of the pelvic floor muscle function (PFMF) in children can be defined based on the results of the study in clinically relevant subgroups, according to the international continence society (ICS) criteria.

### Ethical approval

This study was registered in the Netherlands National Trial Register (NL30551.068.09). The Medical Ethics Committee of the Maastricht University Medical Centre has approved the study. This study has been designed in accordance with the Helsinki Accords and the Dutch Medical Research Involving Human Subjects Act (WMO).

### Framework of the study

**Figure 1:**
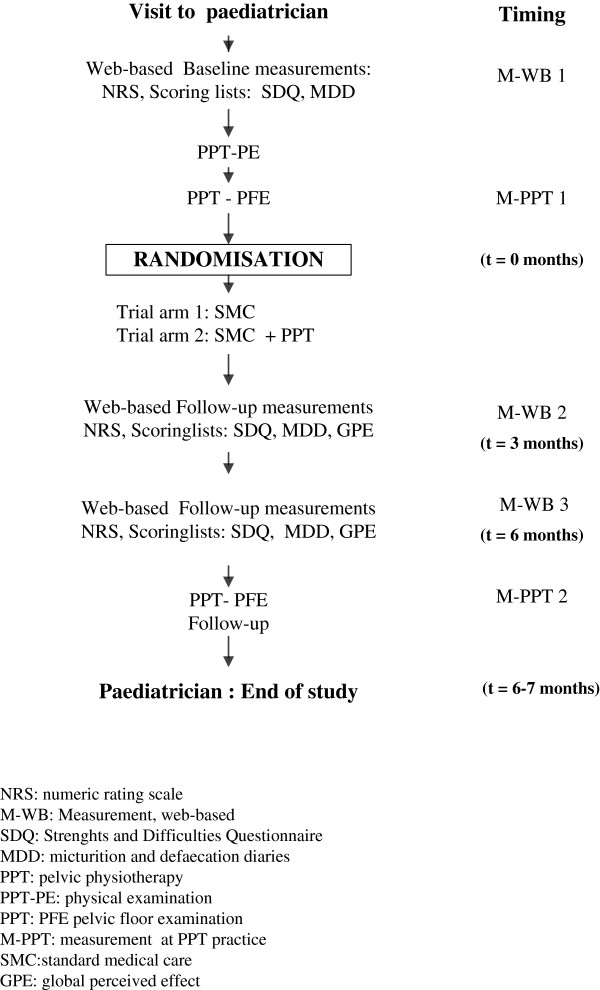
Flowchart of the randomised controlled trial.

**Figure 2:**
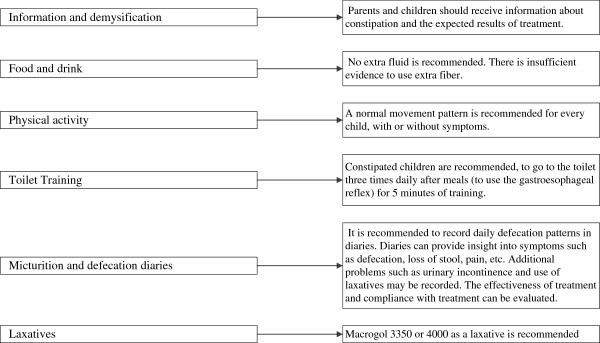
Standard medical care in accordance with the ‘Dutch guideline on constipated children’.

A child’s participation to the study starts and ends with a visit to a paediatrician. In our multicentre randomised controlled trial, the control group will receive only standard medical care (SMC) (Figure 1) whereas the experimental group will have SMC supplemented with PPT (Figure 2). The control group will have at least three contacts with the paediatrician in five months while the experimental group will have a maximum of six contacts with the physiotherapist. The study duration will be six months from randomisation, with a three-year recruitment period.

### Measurement and timing

Web-based measurements will be obtained before the first visit to the physiotherapist (M-WB 1), three months after randomisation (M-WB 2) and before the final visit to the physiotherapist (M-WB-3) (Figure 1). Shortly before randomisation (M-PPT 1) and at six month, adjacent to M-WB 3, measurements at the physiotherapist will take place (M-PPT 2). After M-PPT 2 a final visit at the paediatrician is scheduled.

Web-based measurements include a structured patient reported outcome, which assesses the presence of the Rome III criteria and laxative use, co morbidity (such as urinary problems and abdominal pain), the Strength and Difficulties Questionnaire (SDQ), the numeric rating scale (with regard to experienced burden) and a two weeks diary. At follow-up (M-WB 2 and M-WB 3) the web-based measurements are supplemented with the global perceived effect (GPE).

At the last visit at the paediatrician, use of laxatives and the presence of Rome-III-criteria (primary outcome) are recorded.

### Participants

#### Study population

Children living in the Netherlands and referred to pelvic physiotherapy by paediatricians from various hospitals will be enrolled in this study.

#### Inclusion criteria

(1) age 5–17 years, (2) functional constipation according to the Rome III criteria, (3) attending regular schools, (4) parent(s) sign(s) an informed consent, (5) children aged over 11 years sign an informed consent themselves, and (6) the child and parent(s) are motivated to participate in the study.

#### Exclusion criteria

(1) prior physiotherapy because of defecation or micturition complaints, (2) urotherapy by another professional (nurse, psychologist etc.) at the start of the study, (3) severe delay in motor skills development, (4) endocrine and metabolic disorders (hypothyroidism, hypercalcemia, diabetes mellitus, diabetes insipidus), (5) neurological and psychiatric disorders (spina bifida, cerebral palsy, anorexia nervosa, autism or PDD-NOS), (6) Down Syndrome, (7) Hirschsprung disease, (8) at least 14 points on the Strength and Difficulties Questionnaire, (9) drug-induced constipation and (10) intestinal surgery (except appendectomy).

### Interventions

#### Standard medical care

Standard medical care, provided by paediatricians, will be given in accordance with the Dutch guideline for constipated children (Figure 2) [18]. Essentially, standard medical care consists of education and demystification about the complaint, food, and drink and physical activity as well as toilet training and the use of diaries and laxatives [18]. Usual care laxative use in children with faecal impaction and older than two years is 1–1.5 g /kg per day for a maximum of seven days. The maintenance dose is 0.3 to 0.8 g/kg per day [18]. The dose will be tailored to individual needs during the trial with guidance of the Bristol Stool Form Scale [31].

#### The Dutch pelvic physiotherapy protocol

**Figure 3:**
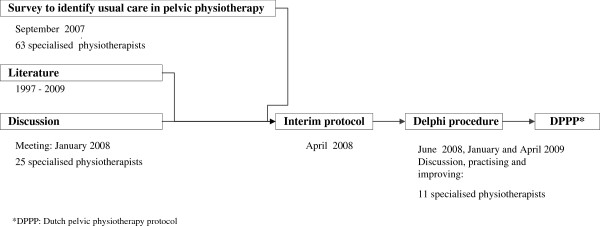
Development of the Dutch pelvic physiotherapy protocol for functional constipation in children.

**Figure 4:**
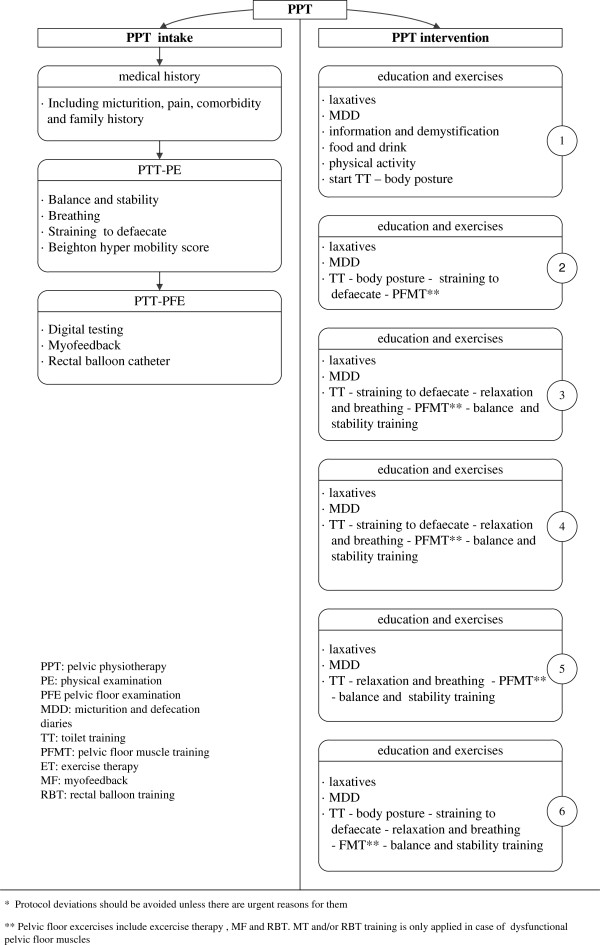
Dutch pelvic physiotherapy protocol’*.

In order to standardise pelvic physiotherapy (PPT) for these children, a survey was first held among 63 Dutch specialised physiotherapists. Extensive agreement existed on providing information, the use of diaries, toilet training, pelvic floor muscle training and biofeedback. On the basis of the survey a provisional PPT protocol was designed in consultation with 25 physiotherapists who were experienced in treating constipated children, using the data from the survey and the literature (Figure 3). Next, the Delphi method was used to discuss, improve and practise the interim protocol during three meetings with all participating physiotherapists. The resulting Dutch pelvic physiotherapy protocol (DPPP) (Figure 4) provides standardisation of intake and intervention.

In this study, according to the DPPP, the PPT intake will consist of medical history tracking, physical examination of at least balance and stability according to M-ABC-2, [24,32,33] assessment of breathing and straining [21,22,25,26], the Beighton hyper mobility score [34,35] and assessment of the pelvic floor muscles and their function [12,29].

The PPT intervention, with a maximum of six sessions will include standard medical care (education, demystification, toilet training, use of diaries and guiding the use of laxatives without intervention of the paediatrician and pelvic floor muscle training using exercise therapy, myofeedback (MFB) and rectal balloon training (RBT), as well as attention to breathing, relaxation and awareness of body and body posture. Awareness of urge and coordination of the PFM during filling and straining will be practised. During MFB, the child will receive information about the activity of the pelvic floor muscles by means of a visual display or a beeping sound. Children with an insensitive rectum will be taught to excrete smaller volumes and to repeat this action until a more normal level of sensory threshold is reached. In the case of an incorrect straining technique, RBT can be applied to learn to strain adequately.

The PPT will be patient-tailored and the sequence and intensity of PPT depends on the child's age and motivation, parents` motivation, co-morbidity and cognition. Toilet training, including pelvic floor muscle training, will be the basis of the PPT and requires considerable attention. All exercises, materials and methods will be presented in a playful manner and in accordance with the children’s age, loco motor skills and perceptions [24,28,29,36].

#### Primary outcome measure

The primary outcome measure is the absence of FC according to the Rome III criteria. The use of less than 10 g of macrogol 3350 or 4000 a day, as a maintenance dose, is allowed.

#### Secondary outcome measures

Numeric rating scale

The two single-item 1–10 numeric rating scales (NRS) measure perceived severity of the problem by the parents and estimate the child’s experience [37].

Strength and Difficulties Questionnaire (SDQ).

The validated SDQ has two versions, one for parents (7–19 years) and one for children (11 years and over), which were developed using the ‘diagnostic and static manual of mental disorders classification’ for psychosocial problems, as these problems (anxiety, depression, behavioural disorders and attention deficit hyperactivity disorders) are relatively common among young people. The total score ranges from 0 to 40 points, with established cut-off points at 0–10 (normal), 11–13 (borderline, presence of less serious issues) and above 14 (high) [38-40].

Global perceived effect

The global perceived effect (GPE) instrument is a subjective score that simply shows the perceived change in symptoms during and after treatment, ranging from 1 (much worse) to 9 (complaints have disappeared). The GPE will be administered at MT-WB 2 and MT-WB 3 before the final visit to the physiotherapist.

Pelvic floor muscle function

The following measurements will be done by the physiotherapist at the second (MT-PPT 1) and final visit (MT-PPT 2).

• Digital testing

• Digital testing will be used to measure the ability and reflex action of the pelvic floor muscles (PFM) at rest and during contraction, cough and straining to defecate. The tone of the internal and external anal sphincters and puborectalis muscle will be palpated, and the sensitivity, anal reflex and presence of stool evaluated. Strength, endurance, coordination and the ability to close the rectum will be evaluated by asking the child to contract and relax the PFM, and then to hold the contraction for 30 seconds. At the same time, the pelvic physiotherapist will assess whether the child is contracting the extra-pelvic muscles, like the abdominal, gluteal and adductor muscles.

• Myofeedback

• Myofeedback uses the electromyographic (EMG) signal of the pelvic floor muscles. The level of the EMG signal should increase while contracting, whereas the signal should decrease at relaxation [15,17,41,42]. Given the absence of an assessment instrument to define proper muscle tone of children's pelvic floor, parameters for the male adults will be used [28,29,36,43].

• For the purpose of this study, the small intra-anal PelviRing Anal Probe will be used.

• The baseline tone of the puborectalis, the maximum strength, and maximum strength after 30 seconds, the tone during straining, the fast and slow twitch and the ability to relax will be measured.

• Values are considered abnormal when: (1) The resting tone is higher than 5 μV (hypertonic) or lower than 2 μV (hypotonic), (2) the sub maximal strength after 30 seconds is not equal to the resting tone, (3) the tone during straining is over 5 μV, (4) onset over 0.2 seconds, (5) offset over 0.2 seconds, (6) the resting tone after testing is higher than 5 μV and (7) the tone fluctuates.

• Rectal Balloon Catheter

• Rectal balloon training (RBT) will be used to develop awareness of the sensory threshold and to strain in a proper way. A neoprene rectal balloon attached to a syringe will be introduced into the rectum and slowly inflated with air to imitate rectal contents. Next, the child will be requested to excrete the balloon, as it would do during defecation. Attention will be paid to the effect of the pelvic floor muscles on sensory threshold, urge sensation, maximum tolerated volume and straining. Satisfactory (reflex) reaction of the external anal sphincter or overreactions of the pelvic floor muscles and of the extra-pelvic muscles like those of the trunk, legs, arms or head are recorded at first sensation, urge and maximum urge. The method of straining is left to the child. The ability to strain sufficiently to defecate is recorded. Absence of reaction of the external anal sphincter, immediately before or at sensory threshold, urge and maximum tolerated volume, will be considered abnormal, as are overreactions of the pelvic floor muscles or the extra-pelvic muscles at straining**.**

### Power of the study

The sample size of this study has been calculated on the basis of a clinical difference of minimal relevance in terms of proportions. The success rate (absence of FC) of the controls has been set at 60% [30,44-46]. There is no published PPT success rate but it is estimated to be 75%. Based on a two-sided effect, an alpha of 0.05, a power of 80% and an expected drop-out rate of 20%, at least 367 children should be enrolled in this study.

### Randomisation and blinding

Randomisation will be based on a list of random numbers generated by a computer in a 1:1 ratio. Concealed randomisation will take place immediately after the second visit to the physiotherapist. Blinding of practitioners, children and outcome assessor is impossible. However, the investigator and parents are blinded for the web-based and PPT baseline measurements and the diary data.

### Data analysis

After checking for missing data and testing for normality, group differences will be analysed using the Pearson Chi-square test, or likelihood ratio in the case of dichotomous outcomes. Continuous outcomes will be analysed using the independent samples t-test or the Mann–Whitney U test. Adjusted group differences will be analysed using multivariable logistic regression with ordinal variables transformed into dummies, taking into account possible differences in mean number of sessions. Finally, subgroup analyses will be done to establish normal values and observed differences in effectiveness for relevant clinical subgroups.

Data analysis will be done according to the intention-to-treat principle. Characteristics of the study population at baseline will be described. Potential effect modifiers like age and gender [10,14], as well as potential confounders like urinary incontinence, enuresis and abdominal pain will be taken into account [17,47-51]. To assess selection bias, responders and nonresponders will be compared in terms of relevant clinical and demographic variables. Data analysis will be carried out using SPSS version 19.0 (SPSS Inc., Chicago, Illinois). A two-tailed p-value of 0.05 will be considered statistically significant.

## Discussion

Why publish a study protocol? This study aims to show that pelvic physiotherapy in combination with standard medical care is more effective than standard medical care alone in constipated children. To our knowledge, this article is the first to describe the design of an RCT among children with FC to assess the effect of PPT as an add-on to SMC. The Dutch Pelvic Physiotherapy Protocol (DPPP) presented here is based on the available literature, a survey and a Delphi procedure among experts. Exchanging knowledge and expertise with other researchers is important in order to prevent duplicate research. Consequently, it has been decided to publish the study protocol prior to achieving results.

The DPPP was developed between 2007 and 2009. Given that no studies have so far been published on the effect of PPT in childhood constipation [52] and that a repeat survey on standard PPT among 49 Dutch physiotherapists in 2011 did not yield substantial differences compared to the 2007 survey, we feel this protocol is as up-to-date as possible.

In the absence of an evaluation tool, we will use parameters for male adults to define the proper childhood pelvic floor muscle functions. These values are expected to be similar to the children’s, as is also the opinion of the experts and the literature [36].

Confounding in our trial may arise due to the Hawthorne-effect as a result of differences in number of the protocolised contacts. Although it is expected, based on experience, that the mean number of treatment sessions will be equal, adjusted group differences will be analysed.

Biofeedback, as a modality of PPT, is an umbrella term in the literature, covering different types of treatment, e.g. manometry (MT), myofeedback (electromyography feedback) (MFB), rectal balloon training (RBT), functional electric stimulation, flowmetry and ultrasound scan. It is administered in various ways depending on availability, costs, setting (clinic/private, discipline involved [physiotherapist/nurse/medical doctor]) and country. There are also differences in the biofeedback method used (peri-anal surface electrodes, intra-anal probes, anal manometry or RBT), the practitioner administering it (gastroenterologist, paediatrician or psychologist), number of treatments and outcome [53-58]. Furthermore, trials show a substantial lack of quality and harmonisation, resulting in heterogeneous treatment protocols and difficulties when interpreting the findings reported in the literature. Mixed results of BF treatment for constipation have been reported [24,54,57,59,60].

FC caused by pelvic floor dysfunction or delayed colonic transit can manifest itself as a single or combined problem [61]. The behavioural approach, concentrating on toilet training and learning to recognise signals from the body such as the urge to defecate, is important for all constipated children [18]. To date, the relationship between pelvic floor muscles, breathing, trunk control and posture [21,22,25,26] has been largely ignored. Therefore, we feel that PFM training must be included in the treatment and should be the starting point of the therapy, rather than being studied as a single modality.

In order to standardise pelvic physiotherapy and minimise information bias, the treatment protocol has been designed in consultation with all participating specialised physiotherapists, who are experienced in treating constipated children.

As with most physiotherapeutic and medical interventions, the practitioner and patient will not be blinded to the treatment interventions. However, the investigator and patient will be blinded for the web-based and PPT baseline measurements and diary data.

Potential confounders will be the presence of urinary incontinence, enuresis, abdominal pain and the use of laxatives [1,18,47]. The presence of incontinence, enuresis and pain can influence the perception of patients' quality of life or may distort the SDQ and GPE scores.

Patients' compliance with the home toilet training and the use of laxatives will be monitored, as it determines the therapy intensity and, indirectly, the success of the therapy. Finally, the use of laxatives will be taken into account, as it can influence the number of stool events and consistency. The results of this trial will be used to evaluate the proposed Dutch pelvic physiotherapy protocol and to determine normal values for the pelvic floor muscle function in children. These outcomes will serve as recommendations in the guideline on PPT in constipated children, which will be developed using the standard method for clinical practice guideline development of the Royal Dutch Society for Physical Therapy (KNGF).

## Endnote

The web-based medical software had been developed by Fastguide, Bergen op Zoom, the Netherlands.

## Abbreviations

BF: Biofeedback; DPPP: Dutch pelvic physiotherapy protocol; EMG: Electromyography; FC: Functional constipation; GPE: Global perceived effect; ICS: International continence society; KNGF: Royal Dutch Society for Physical Therapy; NRS: Numeric rating scale; M-WB: Measurement, web-based; M-PPT: Measurement at PPT practice; MFB: Myofeedback; PFM: Pelvic floor muscles; PFMT: Pelvic floor muscle training; PPT: Pelvic physiotherapy; RBT: Rectal balloon training; RCT: Randomised controlled trial; SDQ: Strengths and Difficulties Questionnaire; SMC: Standard medical care.

## Competing interests

The authors declare that they have no competing interests.

## Authors’ contributions

MvE substantially contributed to the design of the study and the drafting of the manuscript. MvE, NB, RDB and EB were involved in the design of the study, drafting the manuscript and revising it critically. MB and WV critically revised the paper. All authors have read and approved the final manuscript.

## Pre-publication history

The pre-publication history for this paper can be accessed here:

http://www.biomedcentral.com/1471-2431/13/112/prepub
